# Mentalizing self mind but not others: Self‐reported mentalization difficulties in multiple sclerosis

**DOI:** 10.1002/brb3.3612

**Published:** 2024-07-05

**Authors:** Müge Kuzu Kumcu, Zulal Törenli Kaya, Yasemin Hoşgören Alıcı

**Affiliations:** ^1^ Department of Neurology Lokman Hekim University Ankara Turkey; ^2^ Department of Neuroscience Ankara University Ankara Turkey; ^3^ Department of Psychiatry Başkent University Ankara Turkey

**Keywords:** mentalization, multiple sclerosis, social cognition

## Abstract

**Background:**

Mentalization can be defined as a mental process by which an individual directly or indirectly perceives and interprets one's own and others' behavior, emotions, beliefs, and needs based on designed mental states. Mentalization problems may be linked to remove associative white matter fiber disconnection. Multiple sclerosis (MS) is one of the diseases with white matter lesions. By comparing MS patients with healthy controls, it was aimed to assess whether MS patients' mentalization skills are affected.

**Method:**

This study involved 243 participants (170 healthy controls and 73 patients with MS). All the participants completed a sociodemographic questionnaire and the Mentalization Scale (MentS).

**Results:**

While it was discovered that MentS scores for the dimension of others‐based mentalization (MentS‐O) were statistically lower in MS group, there was no statistically significant difference between the groups in terms of the dimensions of motivation to mentalize (MentS‐M) and self‐based mentalization (MentS‐S) scores.

**Conclusion:**

We may conclude that MS patients have trouble comprehending other people's thoughts. This effect can be one of the causes of MS patients' issues with social cognition.

## Graphical abstract

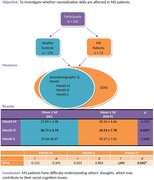

## 1 INTRODUCTION

The concept of mentalization, emphasized in the last decade by Peter Fonagy and colleagues, refers to the way humans comprehend their social world by envisioning the mental states (such as beliefs, motives, emotions, desires, and needs) that underlie both their own and others' behaviors in interpersonal interactions (A. W. Bateman & Fonagy, 2004, 2019; Choi‐Kain & Gunderson, 2008; Fonagy & Allison, 2013; Fonagy & Campbell, 2017; Fonagy et al., 2018). It is the process through which individuals comprehend both others and ourselves, both implicitly and explicitly, in terms of subjective states and mental processes (A. Bateman & Fonagy, 2010). By integrating different schools of thought from the fields of psychoanalysis, developmental psychology, and cognitive neuroscience, Fonagy constructed a theory of how the capacity to mentalize develops in early childhood and, alternatively, how variations from this normal developmental path result in severe forms of adult psychopathology (A. W. Bateman & Fonagy, 2019; Choi‐Kain & Gunderson, 2008; Fonagy & Campbell, 2017; Fonagy et al., 2018). Mentalization is a multi‐faceted concept that incorporates different dimensions, ranging from automatic to controlled mental processes, internally to externally focused perspectives, self to other orientations, and cognitive to affective aspects. Mentalization shares conceptual similarities with empathy, social cognition, emotional intelligence (EI), and theory of mind (ToM). However, it differs from empathy and social cognition in that it encompasses self‐reflection. It goes beyond merely perceiving and understanding emotions (as in EI) or attributing intentions, thoughts, and beliefs (as in ToM). The term theory of mind was introduced into the scientific literature by primatologists observing the chimpanzee's ability to understand an actor's intentions in film clips (Premack & Woodruff, 1978). Research on this particular social cognitive capacity expanded after developmental psychologists introduced the “false‐belief task” (Wimmer & Perner, 1983). ToM refers to mental abilities that allow an individual to first appreciate the existence of different mental states in others and then to accurately identify the mental states of others (e.g., intentions, motives, beliefs, desires, and emotions) in order to interpret their behavior (Choi‐Kain & Gunderson, 2008; Premack & Woodruff, 1978). Mentalization expands upon the ToM by emphasizing a more comprehensive understanding of emotions, interpersonal dynamics, and the development of a stable self‐concept (Choi‐Kain & Gunderson, 2008). Instead, mentalization involves the consideration of various inner states and processes to interpret observable behavior (Dimitrijević et al., 2018). By developing the concept of mentalization, Fonagy applied its internally or self‐oriented and affectively rich qualities to the ToM, which is more empirically developed, outwardly or other‐oriented, and cognitively focused (Choi‐Kain & Gunderson, 2008).

Alexithymia is another concept that is associated with mentalization, especially with self‐mentalization. While mentalization is the capacity to refer to the mental states of others in order to predict their conduct, alexithymia is typified by deficiencies in awareness and differentiation of emotional states (Subic‐Wrana et al., 2011). Mentalization also differs from alexithymia by involving processes related to the management and modulation of emotions (Calaresi & Barberis, 2019).

Mentalization necessitates a wide range of intact cognitive skills that allow people to conceive mental states with plausibility, flexibility, and complexity, but it best connects this cognitive domain of reason and insight with emotion. The integration of cognitive and affective components of both the process and content of understanding mental states enables individuals to “feel clearly” and improves “emotional knowledge” (Allen, 2006).

Mentalization is strongly linked to attachment quality, with securely attached individuals showing better mentalizing abilities than those with insecure attachments. In other words, a nurturing, emotionally connected bond between infant and caregiver supports the formation of secure attachment and creates an environment conducive to developing optimal mentalization skills. Conversely, when caregivers are unresponsive or insensitive to the child's needs for protection and support, it can lead to insecure attachment and impede the development of mentalizing abilities (Fonagy & Luyten, 2018). Signs of impaired mentalization include disinterest in mental states, a lack of imagination about others' mental worlds, a focus on external factors, and a diminished awareness of the connection between internal and external realities (Fonagy & Target, 2008). Several psychiatric disorders, such as depression (Fischer‐Kern & Tmej, 2019), anxiety disorders, post‐traumatic stress disorder, obsessive‐compulsive disorder (Sloover et al., 2022), eating disorders (Simonsen et al., 2020), and borderline personality disorder (A. Bateman & Fonagy, 2010), exhibit impaired mentalization skills. Since mentalization is an important concept in terms of close relationships and psychopathology, it is also an important aspect in psychotherapy and treatment (Allen et al., 2008).

It is widely acknowledged that the temporoparietal junction, the precuneus, and the medial and inferior‐lateral parts of the prefrontal cortex make up this highly distributed neuro‐cognitive network, even though the specific brain regions involved in mentalization are still a matter of discussion. With some degree of right‐hemisphere dominance, this sort of frontotemporoparietal system has been frequently supported by group‐based assessments in neuropsychological populations (Happé et al., 2001; Shamay‐Tsoory et al., 2009; Winner et al., 1998). Complex, distributed neural networks are believed to be the basis for high‐level cognitive functions (Bressler & Menon, 2010; Mesulam, 1998). Cognitive disorders may be brought on by a neurocognitive network's general dysfunction brought on by a loss of structural connectivity (He et al., 2007). Associative white matter fiber disconnection has been linked to reports of mentalization impairment (Herbet et al., 2014).

One of the first diseases that comes to mind when white matter lesions are mentioned is multiple sclerosis (MS). During the course of persistent demyelination, MS patients typically have social and cognitive deficits (Bora et al., 2016; Chiaravalloti & DeLuca, 2008). Impairments in social cognition are a severe precursor to psychiatric comorbidities and reduce the quality of life (Bouchard et al., 2008; Chalah & Ayache, 2017). One of the most critical components of social cognition is the capacity to mentalize. In the literature, the ToM, which aims to understand the mind of others, has been frequently used in studies on MS patients. A recent meta‐analysis showed that patients with MS exhibit moderate impairment in the broad constructs of ToM and empathy and in the subcomponents of ToM (cognitive ToM and affective ToM/cognitive empathy), but no significant impairment in affective empathy. Nevertheless, the cross‐cutting points in the definitions create difficulties in addressing the studies. There is a lack of studies on understanding the thoughts and feelings of self and others and measuring the effect of this on interpersonal relationships. The concept of mentalization seems to be a suitable candidate to fill this gap. Neuroimaging studies also support the possibility of impairment in social cognition.

Abu‐Akel and Shamay‐Tsoory's approach provides a precise description of brain areas involved in ToM (Abu‐Akel & Shamay‐Tsoory, 2011). The model represents the primary brain networks involved in mentalization, taking into consideration neuroscientific data from clinical and non‐clinical populations. According to this model, the temporoparietal junction, superior temporal sulcus, precuneus, and posterior cingulate cortex compose the Core Theory of Mind Network, which allows for the depiction of agency toward mental states (Isernia et al., 2020). The amygdala, ventral striatum, ventral temporal pole, and ventral anterior cingulate cortex (ACC) reflect emotional states of mind, whereas the dorsal striatum, dorsal temporal pole, and dorsal ACC represent cognitive states of mind. Although this model includes a landmark of ToM neural hub components, it is still unclear which brain mechanisms are responsible for mentalizing deficits in pathological conditions (Duclos et al., 2018; Elamin et al., 2012; Trojsi et al., 2016), particularly the interaction between the various social brain areas. The underlying mechanisms related to social cognition and ToM pathologies in MS patients are thought to be due to both gray matter (GM) atrophy (Batista et al., 2017; Chalah & Ayache, 2017; Ciampi et al., 2018; Mike et al., 2013; Pitteri et al., 2019) and white matter (WM) damage (Batista et al., 2017; Isernia et al., 2020; Mike et al., 2013). In MS patients, atrophy in GM has been described at both the global and local levels, and it has been linked to deficiencies in ToM. The volume of the amygdala (Batista et al., 2017; Pitteri et al., 2019) and the cingulate cortex (Chalah & Ayache, 2017) appear to play a significant influence in social cognition abilities and ToM. In contrast, WM injury characterized by widespread microstructural disruption and anatomical disconnection has been linked to poor ToM performance (Batista et al., 2017; Isernia et al., 2020). ToM scores were observed to correlate with normal‐appearing WM microstructural indices of the corpus callosum, fornix, tapetum, uncinated fasciculus, left inferior cerebellar peduncle, and right superior temporal gyrus (Batista et al., 2017). Similarly, Isernia et al. (2020) discovered a significant relationship between cognitive ToM and normal‐appearing microstructural indices in tracts connecting major ToM GM areas (Isernia et al., 2020). In a study investigating self‐other discrimination in MS patients, they pointed out that a decrease in GM volume along the putamen, thalami, and anterior insula, predominantly in the left hemisphere, is associated with less imitation and weaker perspective taking, without a decrease in emotional recognition, and may cause a cognitive self‐bias when confronted with contradictory self and other representations. They interpreted their findings as suggesting that impaired self‐other discrimination and the associated subcortical GM atrophy pattern may underlie the socio‐cognitive impairments observed in MS (Czekóová et al., 2019). In the light of this literature, it can be concluded that many different lesions seen in MS may lead to various skill deficits that may affect the ability to mentalize.

In this study, we aimed to address the ability to understand both one's own mind and the mind of others by considering mentalization holistically. We hypothesized that MS patients would have lower mentalization skills than healthy controls.

## 2 METHODS

### 2.1 Procedure

We conducted a study involving patients diagnosed with MS admitted to the neurology outpatient clinic of Lokman Hekim University. A total of 73 MS patients and 170 healthy controls participated in the study. Healthy controls were selected from the non‐clinical population. To recruit healthy controls, an announcement was made at the psychiatric clinic of a university hospital in Ankara. Data collection involved employing the snowball method, and gathering information from healthcare professionals, students, and companions of patients who applied to the clinic. Those with neurological disease, psychiatric disease, chronic diseases such as renal failure or cancer, head trauma with at least 30 min of altered consciousness, and alcohol/substance misuse, were excluded. It was checked whether participants with low education level (5 years and below) could read or write. All participants completed a sociodemographic form and the Mentalization Scale (MentS).

In the MS group, participants over the age of 18 and with an expanded disability status scale (EDSS) score of 5.5 and below were included in the MS group of the study. Those with dementia, psychiatric disease, chronic diseases such as renal failure or cancer, head trauma with at least 30 min of altered consciousness, and alcohol/substance misuse, were excluded. The EDSS scores were calculated, and information regarding the duration of disease, number of attacks, and medical therapy usage was recorded.

All participants provided informed consent. The Research Ethics Committee at Lokman Hekim university granded ethics committee approval (2023106‐ date: 13.06.2023‐2023/105).

### 2.2 Measures

#### 2.2.1 Mentalization scale

It is a self‐report scale developed by Dimitrijević et al. (2018) to assess mentalization. The scale includes 28 items each with a Likert scale rating of 1−5 (1–completely inaccurate, 5–completely correct). It is broken down into three dimensions: motivation to mentalize (MentS‐Motivation, MentS‐M), others‐based mentalization (MentS‐Others, MentS‐O), and self‐based mentalization (MentS‐Self, MentS‐S). The MentS‐M dimension is designed to assess the intention to comprehend the reason behind both one's own and others' behaviors and emotions (e.g., “I find it important to understand reasons for my behavior,” “ I often think about other people and their behavior”). The MentS‐O dimension aims to evaluate the capacity to perceive the internal states and observable behaviors of other individuals (e.g., “Usually I can recognize what makes people feel uneasy,” “I can make good predictions of other people's behavior when I know their beliefs and feelings”). The MentS‐S dimension includes items related to ability to understand one's own intentions, feelings, and behaviors (e.g., “When I get upset, I am not sure whether I am sad, afraid, or angry,” “I am often confused about my exact feelings”). A Turkish adaptation study of MentS was conducted by Torenli Kaya et al. (20223). The Cronbach's alpha internal consistency coefficient of the Turkish version was reported as 0.84 for MentS total score, 0.78 for MentS‐S, 0.80 for MentS‐O, and 0.79 for MentS‐M (Törenli Kaya et al., 2023).

#### 2.2.2 Expanded disability status scale

The EDSS is a standardized measurement tool used to assess the severity of disability in patients with MS. It quantifies disability based on neurological examination findings and measures various aspects of functioning, such as mobility, coordination, sensory functions, and more (Kurtzke, 1983).

The EDSS scale ranges from 0 to 10, with half‐point increments, where higher scores indicate greater disability. The categorization of EDSS scores is as follows:

EDSS 0.0–1.5: No or minimal disability, fully ambulatory.
EDSS 2.0–3.5: Moderate disability, some difficulty with walking and coordination.
EDSS 4.0–5.5: Significant disability, requiring assistance or aids for walking.
EDSS 6.0–7.5: Severe disability, needing assistance with daily activities.
EDSS 8.0–9.5: Very severe disability, largely or completely confined to bed or wheelchair.

### 2.3 Statistical analysis

IBM SPPS Statistics version 26.0 was used. Statistical significance was determined as *p* < .05. The mean, standard deviation, median, minimum, and maximum scores, and absolute and relative frequencies were all examples of descriptive statistics. To check for statistically significant differences, the Mann–Whitney *U* test was used, depending on whether the data met the assumptions about normal distribution. The χ test was employed to assess the association between the two categorical variables within the MS and HC groups to address between MentS dimensions, Spearman's correlation was used. Bonferroni correction was applied to significant correlations. The effects of age, gender, and education level were analyzed with multivariate logistic regression analysis.

## 3 RESULTS

### 3.1 Demographics and clinical characteristics

A total of 243 participants were included in our study, including 73 MS patients and 170 healthy controls. The descriptive information's of the participants according to age, gender, marital status, education level, and characteristics of the disease for MS group are shown in Table 1.

**TABLE 1:** Demographics and clinical description of people with multiple sclerosis and healthy controls.

	MS *n* = 73	HC *n* = 170	*p* value
**Demographic characteristics**			
**Age** Mean ± SD Median Min‐Max	38.21 ± 10.05 36 21–75	36.23 ± 5.66 35 28–63	.197^a^
**Gender** Female *n* (%)	46 (63)	98 (57.6)	.435^b^
**Marital status** *n* (%) Married Single	53 (72.6) 20 (27.4)	85 (50) 85 (50)	.001^b^
**Education** Mean ± SD Median Min‐Max	13.39 ± 2.71 14 8–20	13.91 ± 1.68 14 5–16	.070^a^
**Clinical features**			
**MS type** PP‐MS RR‐MS SP‐MS	9 (12.3) 61 (83.6) 3 (4.1)		
**Number of recurrences** Mean ± SD Median Min‐Max	2.80 ± 3.17 2 0–20		
**Disease duration** Mean ± SD Median Min‐Max	9.2 ± 5.83 8 1–22		
**EDSS** Mean ± SD Median Min‐Max	1.62 ± 1.31 1 0.5‐5.5		
**Ments dimensions**			
**MentS‐M** Mean ± SD Median Min‐Max	28.10 ± 4.66 28 16–37	27.89 ± 3.98 28 12–35	.959^a^
**MentS‐O** Mean ± SD Median Min‐Max	34.53 ± 7.78 35 9–45	36.77 ± 5.74 37 9–45	.029^a^
**MentS‐S** Mean ± SD Median Min‐Max	29.27 ± 7.01 29 13–45	29.66 ± 8.37 31 12–45	.608^a^

Abbreviations: HC, healthy control; EDSS, expanded disability status scale; MentS‐M, MentS‐Motivation; MentS‐O, MentS‐Others; MentS‐S, MentS‐Self; MS, multiple sclerosis; PP‐MS, primer progressive multiple sclerosis; RR‐MS, relapsing remitting multiple sclerosis; SP‐MS, seconder progressive multiple sclerosis.

^a^ A Mann–Whitney *U* test

^b^ χ test.

### 3.2 Mentalization

The mentalization dimensions are shown in Table 1. While the MentS‐O score was found to be significantly lower in the MS group, no significant difference was found between the two groups in the MentS‐M and MentS‐S scores. The results of multivariate analysis are shown in Table 2.

**TABLE 2:** Multivariate logistic regression analysis between two groups for MentS‐Others (MentS‐O) Score.

	*p* value	Odds ratio
Age	.109	1.032
Gender	.305	1.357
Education level	.119	0.899
MentS‐O	.025	0.953

In the MS group, an inverse correlation was found between EDSS scores and MentS‐O scores. But it disappeared with the Bonferroni correction. No correlation was found between age, education year, disease duration, number of attacks, and MentS scores. They are shown in Table 3. In the HC group, no correlation was found between age, education year, and MentS scores. They are shown in Table 4.

**TABLE 3:** Correlation between the MentS scores and demographic, clinical features and EDSS in multiple sclerosis (MS) group.

	MentS‐M		MentS‐S		MentS‐O	
	r	*p*	*r*	*p*	*r*	*p*
EDSS	−0.115	.345	0.021	.865	−0.243	.042* (.126)^**^
Age	−0.058	.369^*^	−0.081	.498^*^	−0.123	.056^*^
Education	0.002	.990^*^	0.063	.598^*^	0.114	.336^*^
Disease duration	−0.011	.929^*^	−0.164	.167^*^	‐0.141	.235^*^
Number of recurrences	−0.048	.684^*^	0.032	.787	‐0.65	.587^*^

Abbreviations: EDSS, expanded disability status scale; MentS‐M, MentS‐Motivation; MentS‐O, MentS‐Others; MentS‐S, MentS‐Self.

*Spearman's correlation test coefficient, **Bonferroni correction.

**TABLE 4:** Correlation between the MentS scores and age and education in heathy control group.

	MentS‐M		MentS‐S		MentS‐O	
	*r*	*p*	*r*	*p*	*r*	*p*
Age	−0.080	.298^*^	−0.088	.460^*^	−0.031	.692^*^
Education	0.160	.037^*^	0.125	.105^*^	0.033	.667^*^

Abbreviations: MentS‐M, MentS‐Motivation; MentS‐O, MentS‐Others; MentS‐S, MentS‐Self.

* Spearman's correlation test coefficient.

## 4 DISCUSSION

In the clinical course of MS, social cognition has an important role in determining both treatment compliance and prognosis. In the present study, we discussed the concept of mentalization, which is a cornerstone for social cognition and may be a predictor for many psychiatric disorders. In our study, it was observed that MS patients' mentalization skills of others were lower than healthy controls.

The long‐range connection has been argued to be crucial for cognition in the literature (Bressler & Menon, 2010; De Benedictis & Duffau, 2011; Knösche & Tittgemeyer, 2011; Mesulam, 1998). The neurocognitive network's cortical synchronization, which is normally protected by the connections of subcortical white matter, is likely related to the underlying pathophysiological mechanism of “progressive disconnection syndrome.” In this regard, research has demonstrated that disruptions in structural connections have a negative impact on cognitive processes (Karnath et al., 2011; Shinoura et al., 2009) and result in significant functional alterations across the network (He et al., 2007). Therefore, impairment of cognitive functions, including regional and remote area functions, can be expected as a consequence of white matter lesions. In a neuroimaging study using the TOM test in MS patients, it has been reported that as the white matter lesion increases, emotion recognition from facial expressions deteriorates, which is a sign of loss of mentalization ability (Mike et al., 2013). According to a study done on individuals with low‐grade gliomas, mentalization became worse as associative white matter fibril damage increased (Herbet et al., 2014). Additionally, it has been observed that global white matter abnormalities in autism, where mentalization deficiencies are most pronounced, are linked to clinical indices of social and communicative functioning (Gibbard et al., 2013). Consistent with the literature, in this study, MS patients had lower mentalization skills than healthy controls.

In studies conducted with ToM tests, which test aimed at predicting the minds of others, it has been reported that MS patients have difficulty in understanding the emotions of others, empathizing, and understanding the intentions, beliefs, and wishes of others (Berneiser et al., 2014; Isernia et al., 2019; Prochnow et al., 2011; Sara et al., 2023). In a recent study, it was reported that MS patients had difficulty distinguishing between self and others. According to this study, when confronted with competing self‐ and other‐representations, there is less imitation and worse perspective taking, which points to a cognitive self‐bias (Czekóová et al., 2019). In our study, there was no difficulty with mentalizing self. This may mean that neural circuits belonging to the self are relatively preserved. In this case, it may be thought that the boundary between self and other is blurred due to impairment in the skills related to the other. Self‐other discrimination has been reported to be seen, especially in relation to GM involvement (Czekoova et al., 2019). It is recommended to examine mentalization skills in relation to the lesion in future studies. Self‐related impairments may also be observed in more extensive lesions. On the other hand, in contrast to autism studies, studying mentalization through lesions in MS will provide more concrete data in understanding mentalization skills and subtypes.

MentS is a concept similar to alexithymia in another respect. The prevalence of alexithymia in non‐clinical control groups ranges from 9% to 17%. In comparison, the prevalence of alexithymia in MS was reported to vary between 10% and 53% in systematic review (Chalah & Ayache, 2017). In the study by Chahraoui et al. (2014) conducted with a French population, approximately 30% of patients with MS exhibited alexithymia (Chahraoui et al., 2014). Conversely, in another study, Bodini et al. (2008) reported a lower prevalence of 13.8% in Italian patients with MS (Bodini et al., 2008). Recent studies conducted with Turkish populations have found that the rate of alexithymia in MS patients can range from 24.2% to 40.8% (e.g., (Seren et al., 2022; Yilmaz et al., 2023). In the present study, the wide range in the prevalence of alexithymia may have prevented us from finding a significant difference in MentS‐S. As discussed in the literature, these discrepancies could be attributed to differences in the clinical characteristics of the samples, cultural differences, or other unknown factors (Chahraoui et al., 2014). Age, depressive tendencies, and cultural differences may also alter the prevalence (Franz et al., 2008; Mattila et al., 2006; Salminen et al., 1999). Moreover, as emphasized in the review of Chalah and Ayache (2017), the lack of healthy controls in most of the studies evaluating the prevalence of alexithymia in MS patients was identified as a limitation. Further studies may benefit from considering these limitations.

Mentalization is one of the cornerstones for both depressive mood and anxiety (Fischer‐Kern & Tmej, 2019). Anxiety and depression are very common psychiatric comorbid conditions in MS patients (Arnett et al., 2002; Boeschoten et al., 2017; Butler et al., 2016; Feinstein et al., 2014). Also, the relationship between mentalization and depression/anxiety has not been thoroughly investigated in MS populations. The few studies that have investigated the relationship between the ToM and anxiety/depression have produced contradictory results, Feinstein et al., 2014; Lenne et al., 2014 (Dulau et al., 2017; Pöttgen et al., 2013). Studies that approach MS patients holistically and address mentalization, emotion awareness, emotion control, and coping will make it easier to comprehend and manage these psychiatric comorbidities, which are by far the most prevalent. Social comprehension and the ability to empathize constitute the foundation of interpersonal interactions. The absence of these skills can lead to difficulties in interpreting social cues, adhering to social norms, and being sensitive to the emotional states of others. Such challenges may result in withdrawal from the social environment, difficulty in making friends, and a general dissatisfaction with social relationships. Individuals with low MentS‐O scores may experience difficulties in understanding the behaviors and emotions of others. This situation can lead to impairments in communication and social behavior, difficulties in human relationships, and, consequently, isolation. In this context, training programs, therapies, or support groups aimed at improving social skills may be crucial for individuals with MS. Such interventions can assist individuals in better understanding social interactions, enhancing their empathy skills, and, therefore, ameliorating their social relationships.

To summarize, difficulties in understanding the minds of others in MS patients may lead to difficulties in finding social support, and social functioning and may cause secondary psychiatric illnesses. So, it may be valuable to assess mentalization skills in the clinical follow‐up of the disease.

This study's use of a cross‐sectional design is the first limitation. There is no information about the premorbid conditions of the patients. Another limitation is that lesions of neuroanatomical regions were not included in the analysis. A longitudinal study with periodic measurements of MS patients will more concretely show the relationship between lesions and mentalization. Also, due to patient restriction in the clinic, imbalanced sample was used. It is recommended to repeat the study with a larger number of patients. Finally, although the MentS scale yields consistent results in studies on the ToM in MS patients, it is ultimately a self‐rating scale. For this reason, especially patients with severe defects in self‐mentalization may not have given a valid response. Reviewing the scale responses according to the patient's clinic is recommended.

## 5 CONCLUSION

The MentS scale can be considered as a practical scale for measuring social cognition. This study shows that the MentS‐O scale is affected in MS patients and negatively correlates with the EDSS score. MS patients reported difficulty in understanding the minds of others. A difficulty in understanding the mind of others, which may also impact personal relationships, may be the basis of social cognition disorder. Interventions to support mentalization seem to be important in terms of decreasing psychiatric comorbidities and increasing the quality of life of the patient.

## AUTHOR CONTRIBUTIONS

**Müge Kuzu Kumcu**: Conceptualization; data curation; methodology; formal analysis; writing—original draft; writing—review and editing. **Zulal Törenli Kaya**: Data curation; methodology; writing—review and editing; writing—original draft. **Yasemin Hoşgören Alıcı**: Conceptualization; data curation; writing—review and editing; formal analysis; writing—original draft; methodology.

## CONFLICT OF INTEREST STATEMENT

All authors declare no conflicts of interest.

### PEER REVIEW

The peer review history for this article is available at https://publons.com/publon/10.1002/brb3.3612

## Data Availability

The data that support the findings of this study are available from the corresponding author upon reasonable request.
